# Brain tumor epidemiology in the era of precision medicine: The 2017 Brain Tumor Epidemiology Consortium meeting report 

**DOI:** 10.5414/NP301066

**Published:** 2017-10-16

**Authors:** Kimberly J. Johnson, Judith Schwartzbaum, Carol Kruchko, Michael E. Scheurer, Ching C. Lau, Adelheid Woehrer, Johannes A. Hainfellner, Joseph Wiemels

**Affiliations:** 1 Brown School Master of Public Health Program, Washington University, St. Louis, MO,; 2 Division of Epidemiology, College of Public Health, Ohio State University, Columbus, OH,; 3 Central Brain Tumor Registry of the United States, Hinsdale, IL,; 4 Department of Pediatrics, Section of Hematology-Oncology, Baylor College of Medicine, Houston, TX,; 5 Division of Hematology-Oncology, Connecticut Children’s Medical Center, Hartford, CT, and The Jackson Laboratory for Genomic Medicine, Farmington, CT, USA,; 6 Institute of Neurology, Medical University of Vienna, Austria, and; 7 Department of Epidemiology and Biostatistics, University of California San Francisco, CA, USA

**Keywords:** brain tumors, epidemiology, precision medicine, cancer

## Abstract

The Brain Tumor Epidemiology Consortium (BTEC) is an international consortium that aims to advance the development of multicenter and interdisciplinary collaborations that focus on research related to the etiology, outcomes, and prevention of brain tumors. The 18^th^ annual BTEC meeting was held in Banff, AB, Canada, on June 27 – 29, 2017. The meeting focused on the intersection between epidemiology and precision medicine, that is, the use of molecular indicators of risk, early disease and prognosis or precision epidemiology. While traditional epidemiologic approaches group large numbers of participants for statistical power, precision epidemiology is founded on the uniqueness and biology of individual disease characteristics. With this in mind, plenary speakers described the molecular heterogeneity of adult and pediatric brain tumors and how those characteristics are currently being used to guide therapy and etiologic research. Rare subtypes and novel mechanisms for recruitment of individuals for research on brain tumors were discussed along with concepts and methodology related to biological and etiologic heterogeneity. The incorporation of relevant molecular classifiers into population registries was emphasized for its role in future research endeavors, ensuring the accessibility of such tools for researchers and clinicians seeking to improve the lives of individuals with brain tumors and those at risk. The next BTEC meeting will be held in Copenhagen, Denmark, in June 2018.

## Introduction 

The Brain Tumor Epidemiology Consortium (BTEC) is an open scientific forum that fosters the development of collaborations between brain tumor researchers that will lead to a better understanding of the etiology, outcomes, and prevention of brain tumors. To undertake its mission, BTEC members mentor junior investigators as well as those who are new to brain tumor epidemiologic research. Founded in 2003 after an initial meeting sponsored by the U.S. National Cancer Institute’s (NCI) Division of Cancer Epidemiology and Genetics (DCEG) and the U.S. National Institutes of Health’s (NIH) Office of Rare Diseases (ORD), BTEC has evolved to become a self-directed consortium with working groups focused on epidemiological evaluation of adult glioma, meningioma, pediatric brain tumors, and on family-based studies of genetic susceptibility. BTEC is a U.S. National Cancer Institute designated consortium and a non-profit 501(c)(3) corporation. 

**Figure 1. Figure1:**
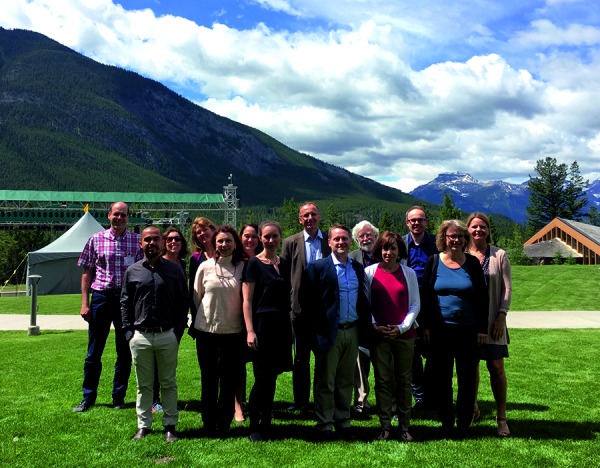
Attendees at the 2017 BTEC meeting in Banff, Canada.

BTEC held its 2017 annual meeting in Banff, Canada, with the major theme “*Brain tumor epidemiology in the era of precision medicine.”* The recent interest in personalized or precision medicine, especially for treating cancer, has brought a renewed interest in the germline and somatic genetic landscapes of the many brain tumor types. While much of this research has been translational at the bedside, discovering new prognostic subgroups and treatment modalities, BTEC sought to define the role that epidemiology would take in formulating the important questions concerning etiology and outcomes in the molecular age of brain tumor research. The program committee included Faith Davis of the University of Alberta School of Public Health in Edmonton, Canada, along with the Board of Director members: Co-Presidents Ching Lau, MD, PhD, and Adelheid Woehrer, MD, PhD; Co-Vice Presidents Johannes A. Hainfellner, MD, and Judith Schwartzbaum, PhD; Secretary Kim Johnson, MPH, PhD; Treasurer Michael Scheurer, PhD, MPH; and past President Joseph Wiemels, PhD. The meeting was coordinated by Ms. Bénédicte Clement of Montpellier, France. The meeting included keynote addresses and a panel discussion with research relevant to the meeting theme. Additionally, there were eleven abstract presentations by junior and senior brain tumor researchers. Researchers from seven countries representing a broad range of disciplines associated with brain tumor research attended the meeting. A summary of the scientific content of the meeting is provided in this report.[Fig Figure1]

## Summary of keynote lectures 

**Daniel Brat, MD, PhD,** of Northwestern University Feinberg School of Medicine, Chicago, IL, USA, gave the first keynote lecture entitled “*A contemporary molecular view of diffuse gliomas with implications for diagnosis”.* Dr. Brat explained that diffuse gliomas are primary central nervous system (CNS) tumors that have been previously classified as astrocytomas, oligodendrogliomas, or oligoastrocytomas and range from World Health Organization (WHO) grades II to IV. Glioblastoma (GBM), grade IV, constitutes 55% of diffuse gliomas in adults. Although diagnoses have historically been based on histopathology, molecular classification has become an established component of diffuse glioma diagnosis. Molecular alterations aid in classification and prediction of both prognosis and therapeutic response allowing for a more refined definition of disease. As a consequence, molecular characteristics are now a required part of WHO classification of glioma. Dr. Brat continued with examples of molecular characteristics that together with histopathology guide glioma diagnosis including isocitrate dehydrogenase (*IDH*) mutations that are frequently seen in grades II and III infiltrating gliomas of adults, as well as secondary GBMs. Primary GBMs typically lack *IDH* mutations and demonstrate *EGFR*, *PTEN*, *TP53*, *PDGFRA*, *NF1*, and *CDKN2A/B* alterations and *TERT* promoter mutations. The mutational spectrum of pediatric high-grade gliomas differs from that in adults with frequent mutations in *H3F3A*, *ATRX*, and *DAXX*, but not in *IDH*. Dr. Brat cautioned that circumscribed, low-grade gliomas, such as pilocytic astrocytoma, pleomorphic xanthoastrocytoma, and ganglioglioma, need to be distinguished from diffuse gliomas in the pediatric population. These gliomas often harbor mutations or activating gene rearrangements in *BRAF*. Although there has been tremendous progress in the molecular classification of diffuse glioma, Dr. Brat emphasized that there is still more work to be done, especially in the area of risk stratification. 

**Sharon A. Savage, MD,** of the U.S. National Cancer Institute gave a talk entitled “*The promise and process of precision medicine in the post-GWAS era*”. Precision medicine takes into account individual genetic, environmental, and lifestyle variability when designing treatments. An example of the benefits of precision medicine includes the case when a rare genetic condition leads to insights important in more common disorders. For example, the rare genetic condition dyskeratosis congenita (DC) is characterized by very short telomeres and an increased risk of certain cancers, bone marrow failure, and many other medical conditions. Germline mutations in the telomere biology gene, *RTEL1,* are one of the causes of DC. Interestingly, genome-wide association studies (GWAS) have independently found common genetic variants in *RTEL1* that are associated with glioma and astrocytoma [1, 2]. Precision medicine can also lead to identifying methods of early cancer detection. Dr. Savage’s group and others found that comprehensive cancer surveillance that includes whole body magnetic resonance imaging (MRI) screening allows for early detection of localized malignancies in patients with Li-Fraumeni syndrome (caused by germline mutation of *TP53*). In addition, precision medicine and prevention calls for an integrated approach to understanding etiology and identifying appropriate treatments. This goal is driving progress in development of analytic methods to quantify interactions among systems (e.g., genetic and immunological interactions). Progress is being made in identifying appropriate treatments given new understandings regarding the complexity of disease causality. 

**Christoph Bock, PhD,** of the CeMM Research Center for Molecular Medicine of the Austrian Academy of Sciences gave a talk entitled “*Dissecting tumor heterogeneity by epigenome mapping and single-cell technologies”.* Dr. Bock discussed three characteristics of cancer epigenetics. First, it complements genetic and transcriptional analysis while addressing some of their limitations. For example, it has been suggested that the sample size required to identify all cancer genes, their interactions and clinical significance, would be larger than that of the world population. In this respect the advantage of epigenomic analysis is that DNA methylation patterns and histone modifications reflect cell states. Cell states are complex dynamic systems but demonstrate relatively few stable states. Using epigenetic biomarkers Dr. Bock’s team was able to identify glioblastoma transcriptional subtypes, predict immune cell infiltration, and observe tumor progression as indicated by DNA methylation of *Wnt* signaling genes. Second, tumor epigenetic heterogeneity is common, relevant, and different from genetic heterogeneity. Epigenome-based reconstruction of cellular differentiation hierarchies provides a framework for analyzing what goes wrong in a diseased organ. For example, in a comparison of genetic and epigenetic heterogeneity between a primary pancreatic tumor and its metastasis, it was noted that there was little heterogeneity in driver mutations between the primary and the metastasis but considerable epigenetic reprogramming in the metastasis. Third, as a result of the advantages described above, frequent monitoring of epigenetic cell states allows the design and use of personalized, adaptive drug combinations. 

**Kimberly J. Johnson, MPH, PhD,** of Washington University in St. Louis, St. Louis, MO, USA, spoke on the topic: “*The promise and perils of international patient registries*”. Dr. Johnson provided background and a summary of her experience as principal investigator of the Neurofibromatosis type 1 Patient Registry Initiative (NPRI). Neurofibromatosis type 1 (NF1) is a rare autosomal dominant disorder affecting an estimated 1/3,000 individuals and is associated with an increased risk for both adult and pediatric brain tumors in addition to other tumors. The NPRI was initiated in 2010 with the objectives of 1) assembling a large patient population with NF1, 2) facilitating research, and 3) applying the information to increase understanding of cancer risk in NF1 that is needed to control health problems in this population. Individuals enroll in the NPRI through a web-based portal and fill out a questionnaire. Dr. Johnson discussed results from different registry evaluations, including a recruitment strategy study where it was reported that of the strategies tested, Facebook and Google advertising yielded the highest number of participants relative to more traditional strategies (e.g., clinic-based recruiting) [3]. With respect to participant characteristics, Dr. Johnson noted that more females enroll than males and participants were from all 50 U.S. states and 49 countries. In addition, she discussed results from a number of other published studies using NPRI data as well as some ancillary studies that have recruited participants through the NPRI. She ended with some perils/considerations of registries where enrollment is based on self-selection including: 1) participation and information biases that can affect the quality/generalizability of the results, 2) keeping up with changing technologies (i.e., smartphone apps), 3) sustainability (funding challenges), and 4) the need for coordination of registries for the same disorder to avoid confusion and duplication of objectives. 

## Panel discussion 

Ms. Carol Kruchko, President of the Central Brain Tumor Registry of the United States (CBTRUS), and Dr. Daniel Brat, the newly appointed Chair, Departments of Pathology, Northwestern University Feinberg School of Medicine and Northwestern Memorial Healthcare, led a panel discussion addressing “*Biomarkers for Cancer Registries: Changing Needs”*. Cancer registries are the source of population-based incidence data on all cases of cancer in a defined geographic area. These registries abide by standards and rules set by organizations known as surveillance stakeholders. In the United States, public laws were passed that mandate cancer data collection including all records of tumors occurring in the brain and other CNS sites using the International Classification of Diseases for Oncology, 3^rd^ Edition (ICD-O-3). CBTRUS works closely with surveillance stakeholders to help ensure that collection practices for CNS tumors result in accurate and complete incidence rates. This role necessitates a close working relationship with neuropathologists responsible for revisions to the classification of these tumors. In June 2016, WHO Classification of Tumors of the Central Nervous System was published by the International Agency for Research on Cancer. This classification system recognized molecular markers for certain brain tumors and other CNS histologies and formulated an integrated histologic/molecular classification. The panel leaders addressed the challenges for cancer registration caused by this new classification and outlined the efforts in progress. In the U.S., collection year and reporting year differ. In 2017, data were reported on all cancer cases diagnosed between 2010 and 2014. This time lag provides an opportunity to address the “changing needs” for future collection practices. Dr. Brat reviewed the history behind the decision to include molecular markers in the 2016 WHO Classification, highlighting the meeting in Haarlem [4] that paved the way for the 2016 WHO molecular classification. Previously, molecular features had been recognized as important diagnostic and prognostic indicators, and the American Joint Commission on Cancer (AJCC) in its 7^th^ Edition Cancer Staging Manual recommended the collection of MGMT methylation and 1p/19q co-deletion along with WHO grade. These data items are collected as site-specific factors in cancer registration with data available from the Surveillance, Epidemiology and End Results (SEER) program [5]. With the support of clinicians, Ms. Kruchko explained that CBTRUS has petitioned the North American Association of Central Cancer Registries (NAACCR) Site Specific Data Items Work Group to include all the biomarkers listed in the 2016 WHO Classification. Another approach was initiated through the ICD-O-3 Revision Committee. It was noted by the panel members that some provisional ICD-O-3 codes assigned to the 2016 histologic/molecular classification are the same codes assigned to the histology only classification (2007 WHO) and, thus, would hinder reporting of these entities by ICD-O-3. This problem has been documented by CBTRUS and submitted to representatives of the National Program of Cancer Registries in preparation for the convening of the ICD-O-3 Revision Committee. Discussion of the rationale behind the code assignments with Dr. David Louis, the lead author of the 2016 WHO Classification, is being planned. The chairs concluded that the challenges with fully implementing the collection of the molecular markers in the 2016 WHO Classification lie with formalizing and submitting requests to the surveillance leadership. Support from established organizations such as BTEC is helpful. The BTEC members present at the meeting unanimously agreed to formally support these efforts. 

**Hubert Caron, MD, PhD,** of Roche in Basel, Switzerland, gave a talk on “*Challenges and opportunities for childhood brain cancer therapies: What are the key targets?*” Developing new therapies for childhood brain tumors has historically taken an excessively long time, partly due to the lack of availability of novel drugs to test in children. Before joining Genentech, Roche, Dr. Caron worked with the European academic consortium Innovative Therapies for Children with Cancer (ITCC) to accelerate the development of novel treatments for pediatric brain tumors. In his talk, Dr. Caron described the innovative Pediatric Oncology Drug Development (iPODD) program at Roche that makes use of cutting edge genomic technologies to rigorously define the molecular targets in various types of pediatric cancers including brain tumors. By utilizing the iMATRIX clinical trial framework that matches targeting agents with validated targets regardless of the tissue origin of the cancer, clinical trials of novel targeted therapy could be made available to pediatric cancer patients at an accelerated pace. Dr. Caron also mentioned another model of academic-industry collaboration, the Innovative Medicine Initiative 2 (IMI2), that is the equivalent of Precision Medicine Initiative in Europe. In addition to describing some of the novel agents that are coming down the pipeline through the various programs, Dr. Caron also mentioned some innovative concepts of drug delivery that could potentially overcome the blood brain barrier and other intrinsic obstacles confronted by physicians in the treatment of brain tumors. 

**Gregory Cairncross, MD,** of the University of Calgary in Calgary, AB, Canada, gave a talk entitled “*Molecular classification of glioma and the quest for precision treatment*”. Diffuse fibrillary glioma (astrocytoma, oligodendroglioma, GBM) has historically been classified by its presumed cell of origin, graded by its microscopic features, which are associated with its growth rate, and treated at a time consistent with its pathology and grade. A paradigm shift in understanding this tumor occurred when The Cancer Genome Atlas Project (TCGA) identified core GBM pathways and gene expression subtypes. Further progress included the classification of glioma by the presence of *IDH* mutations, with very few of these mutations occurring in primary GBM. In addition, methylation subclasses of GBM were also described. Similarly, integrated genomic analyses were conducted for low-grade glioma and oligodendroglioma, the latter tumor being characterized by *IDH* mutations and co-deletion of chromosomal arms 1p and 19q. Unfortunately, the progress in molecular classification has not corresponded to progress in treatment. At present, treatment is essentially the same for all molecular subtypes of diffuse fibrillary glioma. However, in an early example of precision medicine, subgroup analyses of glioma treatment clinical trials showed a survival advantage of 1p/19q deleted tumors when treated with procarbazine, lomustine, and vincristine (PCV) and radiation therapy. In 2005, Stupp et al. [6] developed the present standard of care for glioma which consists of radiotherapy and concomitant adjuvant temozolomide In the same year, Hegi et al. [7] reported that *MGMT* gene silencing predicts response to temozolomide in GBM although MGMT status is not yet used clinically, except as a guide for conducting radiation therapy in elderly GBM patients. Future treatment goals include enhancing temozolomide therapy, thwarting temozolomide resistance, adaptation of immunotherapy to glioma and developing targeted therapy for molecular subtypes. Dr. Cairncross concluded that there is still progress to be made in understanding the etiology and pathogenesis of glioma. 

## Abstract presentations 

There were eleven abstracts presented over 2 days that covered topics ranging from the global descriptive epidemiology of brain tumors to how next-generation sequencing is being utilized in the clinic to uncover germline mutations that may be responsible for tumor development in neuro-oncology patients. The first abstract session began with two junior investigator award presentations that were sponsored by the American Brain Tumor Association. The first junior investigator award presentation was given by **Maral Adel Fahmideh** of the Karolinska Institutet, Stockholm, Sweden. In her talk entitled: “*Common Genetic Variations in Cell Cycle and DNA Repair Pathways Associated with Pediatric Brain Tumor Susceptibility*”, she reported the results of a case-control study that included 245 pediatric brain tumor (PBT) cases and 489 controls from 7 to 19 years old at diagnosis/reference date. 68 single nucleotide polymorphisms (SNPs) in four main pathways including DNA repair, cell cycle, metabolism, and inflammation involved in brain carcinogenesis associated with adult brain tumor susceptibility were genotyped. The study provided evidence for associations between PBTs and SNPs in several cell cycle and DNA repair genes (*EGFR*, *ERCC1*, *CHAF1A*, *XRCC1*, *EME1*, *ATM*, *GLTSCR1*, and *XRCC4*) suggesting that the etiology of pediatric and adult brain tumors may be similar. The second junior investigator award presentation was given by **Adalberto Miranda Filho** of the International Agency for Research on Cancer in Lyon, France. In his talk entitled “*Global epidemiology of brain and central nervous system cancers*”, he described results from a study examining geographic and temporal variations in brain and CNS cancer incidence using 2012 data from the GLOBOCAN database and population-based registries from the Cancer in Five Continents Series (CI5). The main findings of the study included a positive correlation between the Human Development Index and brain tumor incidence and an increase over time in age-standardized rates for brain tumors in several regions of the world, including several South American countries for glioma. 

**Maria Feychting, PhD,** of the Karolinska Institutet in Stockholm, Sweden, gave a talk entitled “*Maternal diabetes and incidence of childhood cancer – A nationwide cohort study in Sweden from 1973 to 2010*”. Dr. Feychting reported results from a population-based cohort study including 3,559,980 children, among whom almost 7,000 were diagnosed with cancer, including ~ 1,700 with PBTs, between 1973 and 2010. The main finding from the study indicated there was an inverse association between maternal diabetes and PBTs. **Beatrice Melin, MD,** of Umea University, Umea, Sweden, gave the next presentation “*Genome-wide association study reveals specific differences in genetic susceptibility to glioblastoma and non-glioblastoma*”. Dr. Melin described the results of a meta-analysis of existing GWAS and a new GWAS from the Gliogene Consortium’s Glioma International Case-Control Study that included over 6,000 cases and 14,100 controls. The study provided further evidence that GBM and non-GBM risk alleles are distinct from each other and underscored that their unique molecular profiles arise through different etiologic pathways. Following Dr. Melin’s talk, **Joseph Wiemels, PhD,** of the University of California San Francisco, San Francisco, CA, USA, gave a talk on “*Maternal cytomegalovirus infections during pregnancy and risk of childhood central nervous system tumors”*. In a cohort study that included individuals born between 1987 and 2010, Dr. Wiemels and his colleagues identified CMV infections through ICD-9 and 10 codes in the Swedish Patient and Medical Birth Registries and children diagnosed with brain tumors before age 15 years in the Swedish Cancer Registry. Dr. Wiemels reported no association between childhood brain tumors and CMV infection prenatally or postnatally. Next, **Adelheid Woehrer, MD, PhD,** of the Medical University of Vienna, Vienna, Austria, gave a talk on “*Patterns of diagnostic marker assessment in adult diffuse glioma: a Survey of the European Confederation of Neuropathological Societies (Euro-CNS)*”. Dr. Woehrer reported the results of a study that surveyed members of the European Confederation of Neuropathological Societies to determine the clinical practices of neuropathologists’ regarding the use of molecular markers in glioma diagnoses and the diagnostic techniques they routinely use. The study results included 130 responses from participants from 40 countries and suggested that neuropathologists view molecular marker testing as highly relevant and have already incorporated molecular information into their diagnostic practice. However, the survey also indicated that there are concerns about the validity of certain tests including those for *MGMT*, 1p19q, and *ATRX*. The final presentation from the first abstract session was given by **David Solomon, MD, PhD,** of the University of California San Francisco, San Francisco, CA, USA. His talk was titled *“Targeted NGS of paired tumor and normal DNA reveals frequent cancer predisposing germline alterations in neuro-oncology patients”.* Dr. Solomon discussed the use of targeted next-generation sequencing in a study of 119 consecutive pediatric and adult neuro-oncology patients with primary CNS neuroepithelial tumors who underwent testing on the UCSF500 Cancer Gene Panel that includes 500 cancer-associated genes. Dr. Solomon’s study revealed several unsuspected germline alterations associated with an increased cancer risk in 21% of patients. This finding has significant implications for patient management as well as for preventive screening of family members harboring the detected alleles. 

On the second day of the conference, four abstract presentations were given. **Faith Davis, PhD,** of the University of Alberta School of Public Health in Edmonton, AB, Canada, spoke about “*Socioeconomic status, urban-rural residence, and brain cancer survival in Canada: 1996 – 2008*”. Dr. Davis reported the results of a brain tumor survival study using Canadian Cancer Registry data for individuals diagnosed with adult primary malignant brain tumors during 6/1/1996 – 12/31/2008. The major finding from this study was that income may have a stronger impact on brain tumor survival than location of residence. This finding may reflect patterns of access to medical care and lifestyle or treatment factors related to the area of residence. Dr. Davis’ talk was followed by a talk from **Yoshitaka Narita, MD**, of the National Cancer Center Hospital in Tokyo, Japan. His talk was entitled “*Survival and clinical characteristics of patients with gliomas from the Brain Tumor Registry of Japan in 2005 – 2008*”. Dr. Narita reported the results from a study including 16,722 primary and 3,200 metastatic brain tumor cases diagnosed from 2005 to 2008 from 116 Japanese institutions. The major finding from this hospital-based study was that most patients with glioma present with low Karnofsky performance status at diagnosis, suggesting that prognosis might improve with earlier diagnosis. 

The final two presentations continued to reflect the wide variation in expertise that the BTEC meetings have traditionally encompassed. **Michael Scheurer, PhD**, **MPH,** of Baylor College of Medicine in Houston, TX, USA, spoke about “*Chromosomally-integrated Human Herpesvirus-6 in familial glioma*”. Using specimens from GLIOGENE, the prevalence of inherited chromosomally-integrated Human Herpes Virus 6 (ici-HHV6) was compared between sporadic (n = 200) and non-syndromic familial glioma cases (n = 195) using quantitative PCR targeting a common HHV6A/B sequence. The major finding from this study was that ici-HHV6 has very low prevalence in glioma (only two cases were observed in familial glioma), and, therefore, it is unlikely that ici-HHV6 is an etiological factor in familial glioma. Dr. Scheurer noted that further studies are warranted to investigate whether ici-HHV6 could act as a modifying factor in families that have a predisposing mutation in cancer-related genes. 

The concluding presentation by **Annette Molinaro, PhD,** of the University of California San Francisco, San Francisco, CA, USA, focused on a promising new statistical methodology. In her talk entitled “*Expanding the predictive ability of an interpretable tree*”, Dr. Molinaro described the results of a methodological study that aims to improve risk prediction and stratification in cancer patients. She and her colleagues developed a prediction model called partDSA that employs a multivariate method to build a decision tree for risk prediction. In the talk, Dr. Molinaro discussed the extension of partDSA that allows harnessing of the predictive ability of a “forest”, or collection of trees, with the interpretability of a single tree. This has direct clinical relevance, as it can provide a robust method for risk prediction with easy clinical interpretability. 

## Conclusions 

The 2017 BTEC meeting covered a diverse set of topics in both adult and childhood brain tumors, but was united by a directed attention on the importance of individual-level molecular classifiers in the research and treatment of brain tumors. Key-note speakers spoke of the value of tumor genetic characteristics on defining prognosis and treatment strategies, including the discovery of new pathways for drug development. The addition of these same classifiers within population registries will allow for new research avenues for brain cancer etiology and prevention. Rare familial cancers can often prove new concepts in disease etiology when new mutations are discovered, and comparisons between common sporadic brain cancers defined by germline polymorphisms or tumor epigenetic pathways reveal common themes in brain cancer development that can be exploited in future research. The 2017 meeting additionally focused on novel and modern methods of study recruitment and data collection with the help of digital media and web-based registries. The molecular complexities of human brain cancers are moving beyond the discovery stage, and are now being applied to improve the lives of those diagnosed and at risk for brain tumors. This was exemplified by the majority of abstract presentations at the 2017 BTEC meeting (6 of 11) being dependent on the use of molecular markers. Clearly, a promising future awaits the *precision epidemiology* of brain tumors. 

## Funding 

We are thankful for the generous meeting support of the American Brain Tumor Association and the National Institutes of Health R13 grant to Joseph Wiemels, PhD. Funding for this conference was made possible (in part) by 1R13CA221308 from the National Cancer Institute. The views expressed in written conference materials or publications and by speakers and moderators do not necessarily reflect the official policies of the U.S. Department of Health and Human Services, nor does mention by trade names, commercial practices, or organizations imply endorsement by the U.S. Government. 

## Conflict of interest 

All authors declare that there are no conflicts of interest.
